# Supplementary material for: Genome-wide analysis of the interplay between chromatin-associated RNA and 3D genome organization in human cells
Source: Nat Commun. 2023 Oct 16;14:6519. doi: 10.1038/s41467-023-42274-7 (PMC10579264; doi:10.1038/s41467-023-42274-7)
Supplement: Supplementary file 1 — Supplementary Information [file 41467_2023_42274_MOESM1_ESM.pdf]

Supplementary Information File

Genome-wide analysis of the interplay between chromatin-associated RNA and 3D genome organization in human cells (Calandrelli R, Wen X, et al.)

Supplementary Figure S1. Distribution of RNA attachment levels (RAL) on the genome. (a) RNA-DNA contact frequency (y axis) vs. the genomic distance between the mapped RNA-end and the DNA-end (x axis) in H1 (blue), HFF (green), and K562 cells (red). (b) The RAL of every TAD (row) and its equal-length flanking regions, based on all the RNAs transcribed from any genomic sequences outside of this TAD (row). The TAD lengths are normalized (center, x axis). Curve at the bottom: average RALs of all TADs. (c-e) Scatterplot of Hi-C's first eigenvector (PC1, y axis) and cRAL (x axis) on every 500 kb genomic bin (dot) of the entire genome in H1 (c), HFF (d), and K562 (e). SCC: Spearman correlation coefficient. (f-h) Scatterplot of gene density (y axis) and cRAL (x axis) on every 500 kb genomic bin (dot) of the entire genome in H1 (f), HFF (g), and K562 (h). The correlation between cRNA and gene density is weaker than the correlation between caRNA and Hi-C's PC1.

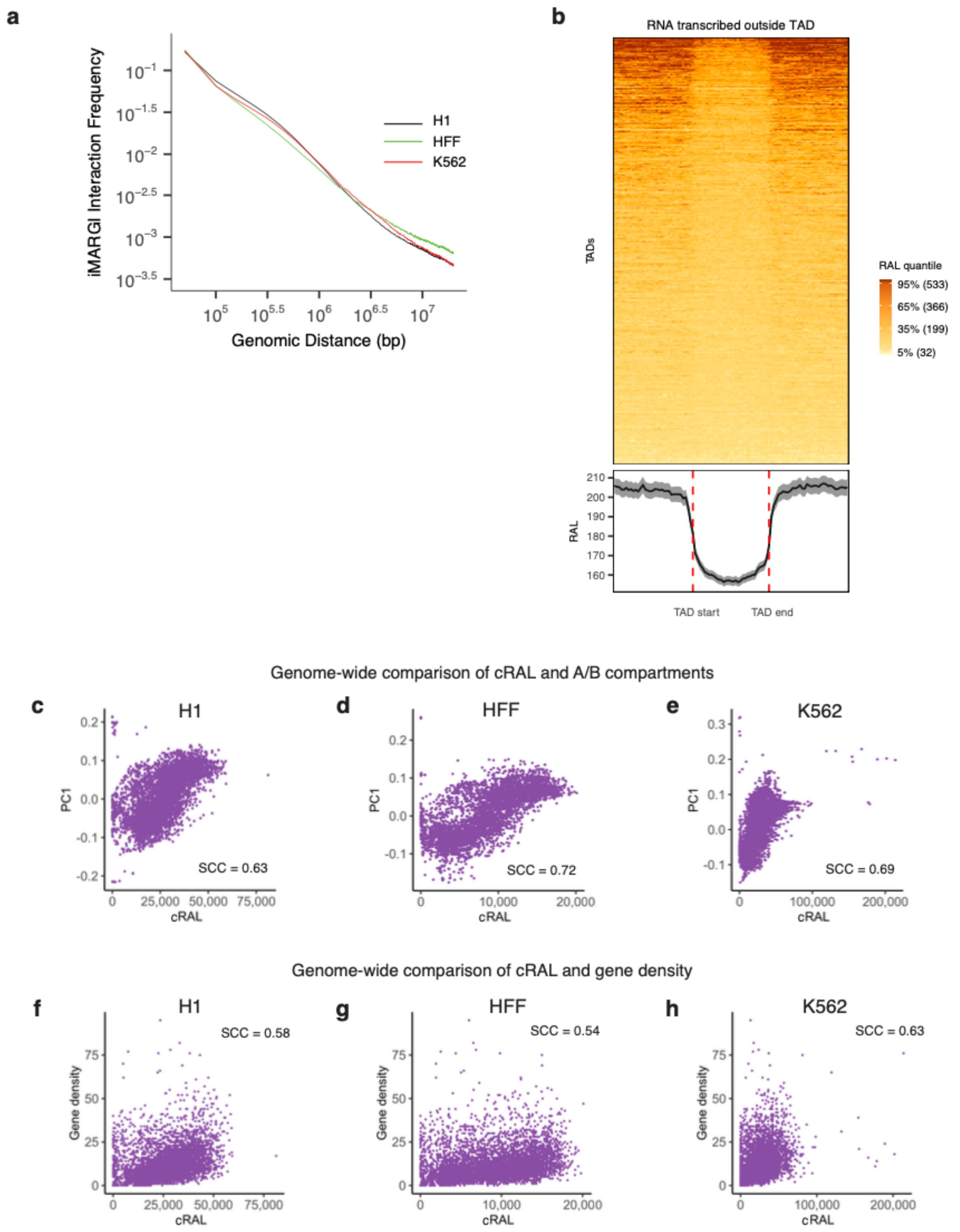

Supplementary Figure S2. Immunofluorescence analyses of SC35 and SON. (a-h) Immunostaining of SON (a-d) and SC35 (e-h) in control, NH4OAc, FL, and RNase-treated H1 cells. Scale bar = 6 µm. (i-k) Distribution of SON's average number of foci per nucleus in control and each treatment (first row). *: p-value < 0.05. **: p-value < 0.01, Wilcoxon test. In comparison, SON's mean background fluorescence (last row) does not change between control (pink) and each treatment (green). ns: not significant. (l-n) Distribution of SC35's average number of foci per nucleus in control and each treatment (first row). **: p-value < 0.01. ***: p-value < 1.0e-3, Wilcoxon test. In comparison, SC35's mean background fluorescence (last row) does not change between control (pink) and each treatment (green). ns: not significant. The upper whisker extends from the hinge to the largest value no further than 1.5 * IQR from the hinge (where IQR is the inter-quartile range, or distance between the first and third quartiles). The lower whisker extends from the hinge to the smallest value at most 1.5 * IQR of the hinge.

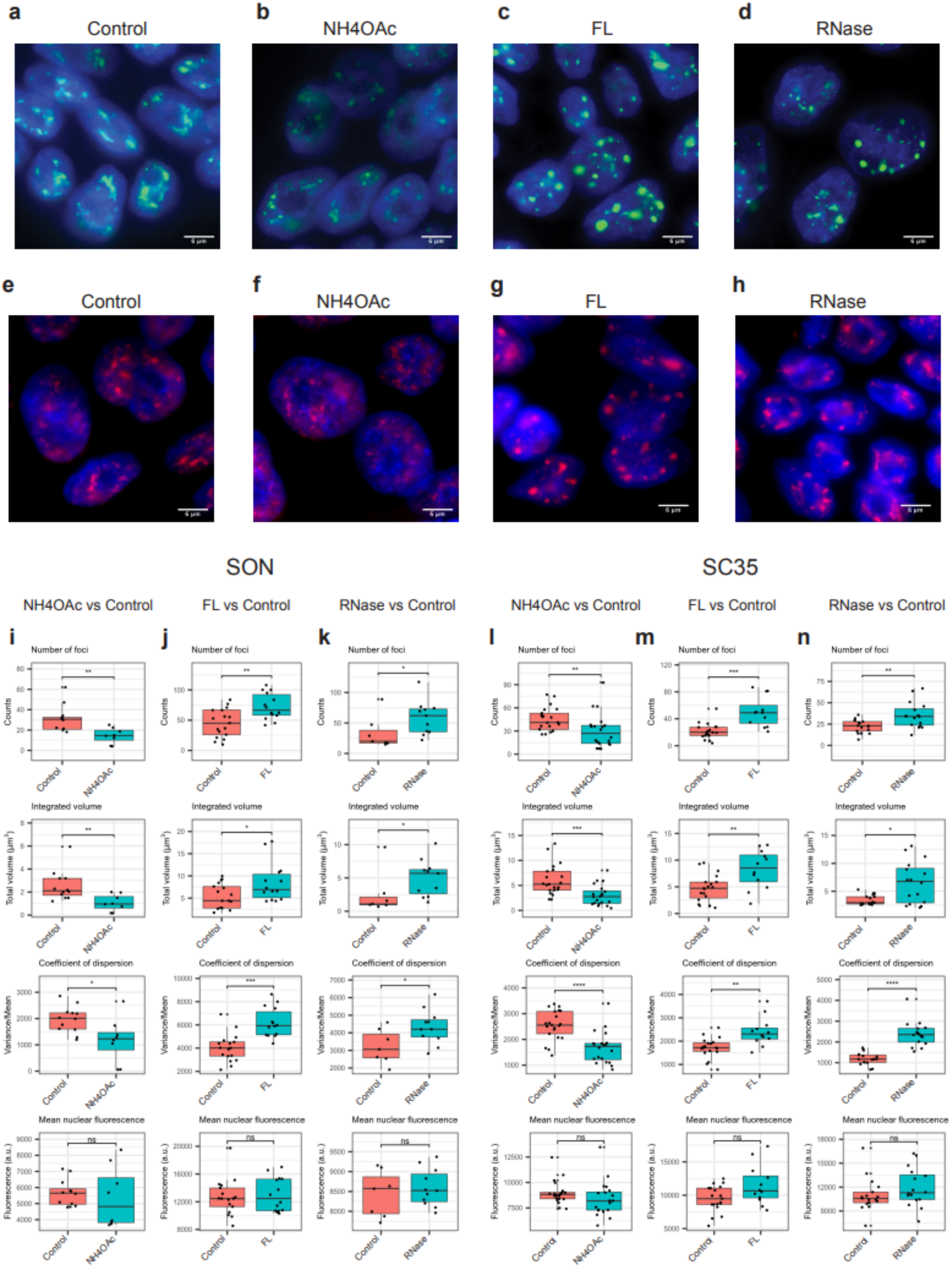

a
Control
b
NH4OAc
c
FL
d
RNase
e
Control
f
NH4OAc
g
FL
h
RNase
SON
SC35
NH4OAc vs Control
FL vs Control
RNase vs Control
NH4OAc vs Control
FL vs Control
RNase vs Control
i
j
k
l
m
n
Number of foci
Counts
Integrated volume
Total volume (μm³)
Coefficient of dispersion
Variance/Mean
Mean nuclear fluorescence
Fluorescence (a.u.)
Control
NH4OAc
FL
RNase
ns

Supplementary Figure S3. An example of loop change. iMARGI contract matrix of control (a), NH4OAc (b), FL (c), and RNase (d) for the corresponding genomic region of Figure 3d. Blue dots: Hi-C derived loops that are superimposed on the iMARGI contact maps. Arrows: a shared loop in FL and RNase that is absent in control and NH4OAc.

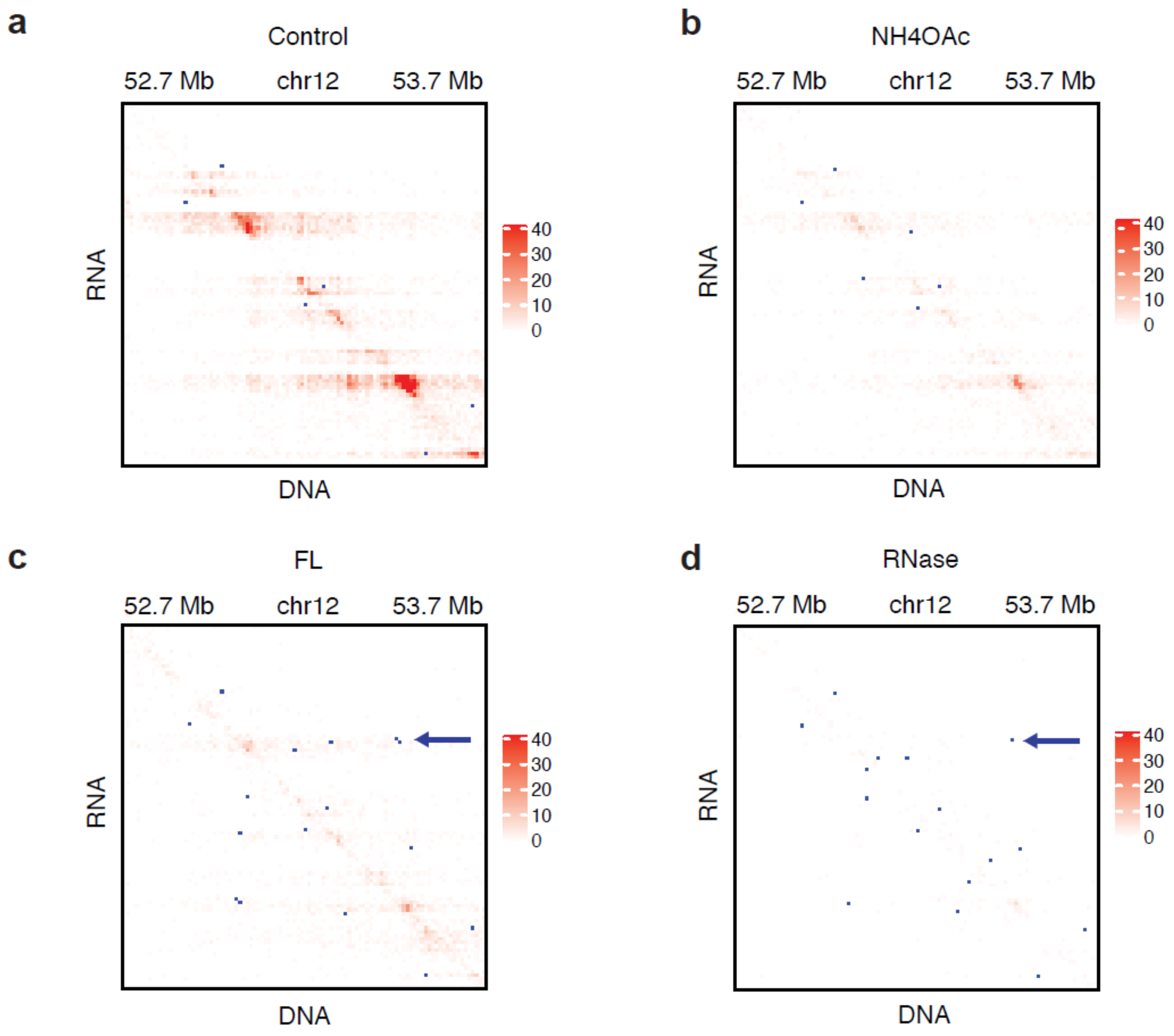

Supplementary Figure S4. RNA-association domains. (a-c) Scatter plots of each RNA-association domain's size (x axis) and the length of the longest gene in each RNA-association domain (y axis). Each dot represents an RNA-association domain, corresponding to a detected rectangular block from iMARGI's contact matrix. The width and the height of each rectangular block correspond to the size of an RNA-association domain and the length of the genomic sequence that produced the caRNA in this domain. The height of each rectangular block often matches the length of the longest gene overlapping with this RNA-association domain, suggesting that most RNA-association domains are decorated by the RNA of single genes. (d) Upset plot of the numbers of RNA-association domains in untreated H1 (Control) and H1 treated with NH4OAc, FL, and RNase. (e) Distributions of the heights of the detected blocks, *: p-value < 1e-25, ***: p-value < 1e-75, ****: p-value <1e-100, Wilcoxon test. (f) Distributions of the widths of the detected blocks. *: p-value < 1e-50, ***: p-value < 1e-180, Wilcoxon test. The center line of the boxplots is the median. The lower and upper hinges correspond to the first and third quartiles (the 25th and 75th percentiles). The upper whisker extends from the hinge to the largest value no further than 1.5 * IQR from the hinge (where IQR is the inter-quartile range, or distance between the first and third quartiles). The lower whisker extends from the hinge to the smallest value at most 1.5 * IQR of the hinge.

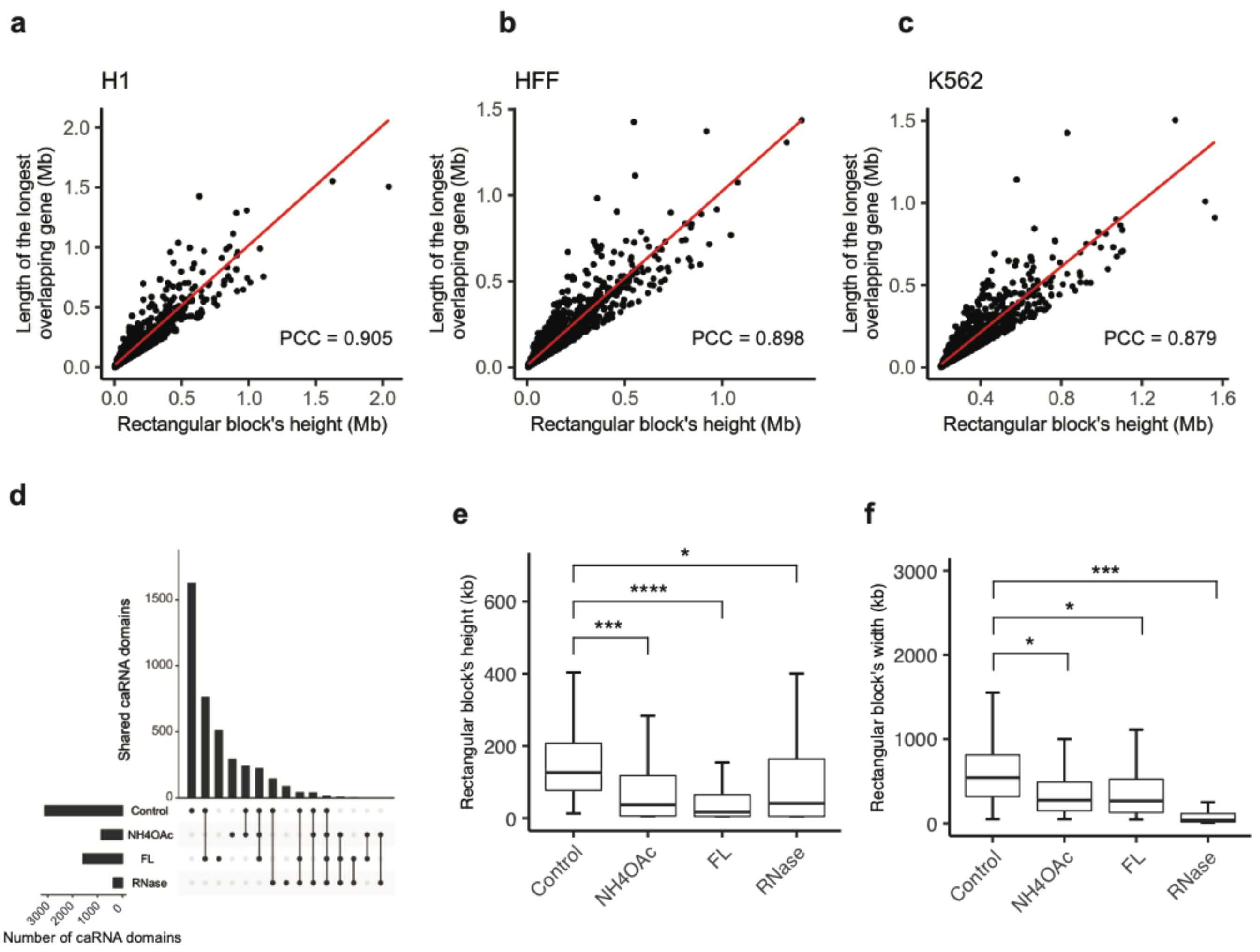

Table S1. Summary of iMARGI and Hi-C datasets.

**a. iMARGI datasets**				
Cell line	Treatment	Number of replicates	Total # of read pairs	Source
H1	None	4	2,642,778,166	This work
	NH4OAc	2	1,247,204,613	
	FL	2	1,438,312,761	
	RNase	2	1,670,230,715	
HFF	None	2	2,755,576,893	
K562	None	2	1,293,950,206	
**b. Hi-C datasets**				
Cell line	Treatment	Number of replicates	Total # of read pairs	Source
H1	None	2	616,625,628	This work
	NH4OAc	2	613,098,350	
	FL	2	604,503,572	
	RNase	2	654,798,738	
HFF	None	2	2,764,855,452	4DNESNMAAN97
K562	None	6	907,136,828	4DNESI7DEJTM
**c. iMARGI datasets in engineered cells**				
Cell line	Treatment	Number of replicates	Total # of read pairs	Source
H9 MLC2v:H2B	None	1	692,140,673	This work
H9 MLC2v:H2B HERV2-KO	None	1	566,783,631	
H9 MLC2v:H2B HERV2-ins-clone2	None	1	967,136,454	
**d. Hi-C datasets in engineered cells**				
Cell line	Treatment	Number of replicates	Total # of read pairs	Source
HCT116 RPB1-Dox-OsTIR1-mClover-mAID	Doxycycline (control)	1	632,233,849	This work
	Doxycycline and 5-Ph-IAA	1	716,607,191	
H9 MLC2v:H2B	None	2	552,532,207	GSE116862
H9 MLC2v:H2B HERV2-KO	None	2	635,909,624	

Extended Text: RBP1 depletion

RBP1, encoded by the POLR2A gene, is the largest subunit of RNA polymerase II. We used the second generation of the auxin-inducible degron (AID2) technology to deplete RBP1[1]. In HCT116 RPB1-Dox-OsTIR1-mClover-mAID cells (RPB1-AID2 cells), where RBP1 is tagged for acute depletion upon addition of doxycycline and 5-Ph-IAA[1]. As the control, we treated RPB1-AID2 cells by doxycycline for 24 hours without 5-Ph-IAA and followed with Hi-C (No-depletion Ctrl), which yielded 632,233,849 read pairs. For the depletion experiment, we treated RPB1-AID2 cells by doxycycline for 24 hours and 5-Ph-IAA for 6 hours and followed with Hi-C (Depletion group), which yielded a comparable number (716,607,191) of read pairs to the IAA- Ctrl. We subjected these data loop calling with HiCCUPS[2]. The Depletion group yielded 3,307 loops, which is approximately 16% more than the detected loops in No-deletion Ctrl (2,619) (p-value = 7.9e-9, paired t-test, chromosome by chromosome). These data suggest depletion of RBP1 led to an increase in loop number.

Taking the union of the loops in the No-depletion Ctrl and the Depletion group, we obtained a total of 5,241 loops (Union loops). We compared the loop strengths (P2LL)[3] between the No-depletion Ctrl and the Depletion group using all the Union loops. The Depletion group exhibited higher P2LLs than the No-depletion Ctrl (fold change = 1.06, p-value < 2.07e-14, paired t-test), suggesting an increase in loop strengths. Taken together, acute depletion of RBP1 resulted in more and stronger chromatin looping.

References

1. Yesbolatova, A. *et al.* The auxin-inducible degron 2 technology provides sharp degradation control in yeast, mammalian cells, and mice. *Nat Commun* **11**, 5701 (2020). https://doi.org:10.1038/s41467-020-19532-z
2. Durand, N. C. *et al.* Juicer Provides a One-Click System for Analyzing Loop-Resolution Hi-C Experiments. *Cell Syst* **3**, 95-98 (2016). https://doi.org:10.1016/j.cels.2016.07.002
3. Rao, S. S. *et al.* A 3D map of the human genome at kilobase resolution reveals principles of chromatin looping. *Cell* **159**, 1665-1680 (2014). https://doi.org:10.1016/j.cell.2014.11.021
