# Supplementary material for: Genome-wide analysis of the interplay between chromatin-associated RNA and 3D genome organization in human cells
Source: Nat Commun. 2023 Oct 16;14:6519. doi: 10.1038/s41467-023-42274-7 (PMC10579264; doi:10.1038/s41467-023-42274-7)
Supplement: Supplementary file 3 — Description of Additional Supplementary Files [file 41467_2023_42274_MOESM3_ESM.pdf]

Description of Additional Supplementary Files

Genome-wide analysis of the interplay between chromatin-associated RNA and 3D genome organization in human cells (Calandrelli R, Wen X, et al.)

File Name: Supplementary Data 1
Description: Sequences of oligonucleotides used in this study. The sequence details of oligonucleotides used in the CRISPRi and 3C-PCR experiments is enlisted. The CRISPRi guides are designed as described in the methods section. The sequences denoted by "N" in the template T7ForVar is replaced by the protospacer sequences.
