# Supplementary material for: Genome-wide analysis of the interplay between chromatin-associated RNA and 3D genome organization in human cells
Source: Nat Commun. 2023 Oct 16;14:6519. doi: 10.1038/s41467-023-42274-7 (PMC10579264; doi:10.1038/s41467-023-42274-7)
Supplement: Supplementary file 5 — Reporting Summary [file 41467_2023_42274_MOESM5_ESM.pdf]

nature portfolio

Corresponding author(s): Sheng Zhong

Last updated by author(s): 9/21/2023

Reporting Summary

Nature Portfolio wishes to improve the reproducibility of the work that we publish. This form provides structure for consistency and transparency in reporting. For further information on Nature Portfolio policies, see our Editorial Policies and the Editorial Policy Checklist.

Statistics

For all statistical analyses, confirm that the following items are present in the figure legend, table legend, main text, or Methods section.

n/a	Confirmed	
☐	☒	The exact sample size (*n*) for each experimental group/condition, given as a discrete number and unit of measurement
☐	☒	A statement on whether measurements were taken from distinct samples or whether the same sample was measured repeatedly
☐	☒	The statistical test(s) used AND whether they are one- or two-sided<br>*Only common tests should be described solely by name; describe more complex techniques in the Methods section.*
☒	☐	A description of all covariates tested
☐	☒	A description of any assumptions or corrections, such as tests of normality and adjustment for multiple comparisons
☐	☒	A full description of the statistical parameters including central tendency (e.g. means) or other basic estimates (e.g. regression coefficient) AND variation (e.g. standard deviation) or associated estimates of uncertainty (e.g. confidence intervals)
☐	☒	For null hypothesis testing, the test statistic (e.g. *F*, *t*, *r*) with confidence intervals, effect sizes, degrees of freedom and *P* value noted<br>*Give P values as exact values whenever suitable.*
☒	☐	For Bayesian analysis, information on the choice of priors and Markov chain Monte Carlo settings
☒	☐	For hierarchical and complex designs, identification of the appropriate level for tests and full reporting of outcomes
☐	☒	Estimates of effect sizes (e.g. Cohen's *d*, Pearson's *r*), indicating how they were calculated

Our web collection on statistics for biologists contains articles on many of the points above.

Software and code

Policy information about availability of computer code

Data collection

iMARGI: Illumina NovaSeq S4 and Illumina HiSeq 4000 were used for sequencing, and Illumina Casava1.8 software for base calling. Data was collected and processed using the iMARGI pipeline (https://sysbio.ucsd.edu/imargi_pipeline). Paired-end read pairs were aligned using bwa mem (0.7.17) with parameter '-SP5M'. pairtools (v0.2.0) and in-house scripts (all available in the iMARGI pipeline) were used to parse, deduplicate and filter the mapped read pairs. Any iMARGI read pair in which the RNA end and the DNA end mapped to within 1,000 bp of each other on the genome are removed from the data analysis.

Hi-C: Hi-C was performed using an Arima-HiC kit (Arima Genomics, Inc., material# A510008, Document# A160134 v00) following the manufacturer's manual. Illumina HiSeq 4000 was used for sequencing, and Illumina Casava1.8 software for base calling. Hi-C data was processed following 4DN consortium's Hi-C data processing protocol (https://www.4dnucleome.org/protocols.html). Briefly, the Hi-C data were processed using the 4D Nucleome (4DN)'s Hi-C Processing Pipeline (v0.2.5) (https://data.4dnucleome.org/resources/data-analysis/hi_c-processing-pipeline), with MAPQ > 30 to filter out multiple mappings.

Data analysis

iMARGI: Data were analyzed using in-house R scripts, exploiting several R packages such as: GenomicRanges (1.38.0), GenomicAlignments (1.22.1), GenomicInteractions (1.20.3), InteractionSet (1.14.0) as the main packages for genomic data manipulation; KaryoploteR (1.12.4) and Gviz (1.30.3), umap (0.2.7.0) for visualization of genomic data and tracks, for ComplexHeatmap (2.2.0) to make heatmaps. For generalized linear regression and LASSO model training and prediction, we used glmnet (4.1.1), ROCR (1.0.11) and MASS (7.3.54). To compute the RAL level at all TAD regions and TAD region rescaling, the software deeptools (3.4.3) was used. caRNA domains were identified using the RNAStripeTools pipeline (https://github.com/Zhong-Lab-UCSD/rnaStripe). The software involved in the RNAStripeTools pipeline are Homer (4.0), bedtools (2.26.0), clodius (0.3) and Python (3.4). In-house Python and shell scripts were used to perform caRNA domains related analysis. The following software and Python libraries were used to perform the analysis: bedtools (2.26.0), samtools (1.8), higlass-manage (0.8.0), matplotlib (3.3.2), numpy (1.19.1), scipy (1.5.2) and pysam (0.15.3).

Hi-C: The output .pairs file were provided to Cooler (v0.8.10) and Juicer Tools (v1.22.01) to generate .mcool and .hic files. The .mcool file was used in HiGlass for visualization. The .hic files were inputted in Juicer Tools for A/B compartment, TAD, and loop analyses. A/B compartments

were called by Juicer's "Eigenvector" tool, TADs were called by Juicer's "Arrowhead" tool, and loops were called by Juicer's "CPU HiCCUPS" tool. Except for the resolution parameter, all the other parameters were left as the default. Data were analyzed using in-house R scripts, exploiting several packages such as: GenomicRanges (1.38.0), GenomicAlignments (1.22.1), GenomicInteractions (1.20.3) as the main packages for genomic data manipulation; KaryoploteR (1.12.4) and Gviz (1.30.3) for visualization of genomic data and tracks, ComplexHeatmap (2.2.0) to make heatmaps.

The codes used for the analysis have been deposited and made publicly available on GitHub at https://github.com/Zhong-Lab-UCSD/RNA3Dgenome-code-repository.

For manuscripts utilizing custom algorithms or software that are central to the research but not yet described in published literature, software must be made available to editors and reviewers. We strongly encourage code deposition in a community repository (e.g. GitHub). See the Nature Portfolio guidelines for submitting code & software for further information.

Data

Policy information about availability of data

All manuscripts must include a data availability statement. This statement should provide the following information, where applicable:

- Accession codes, unique identifiers, or web links for publicly available datasets
- A description of any restrictions on data availability
- For clinical datasets or third party data, please ensure that the statement adheres to our policy

All high-throughput data supporting the current study have been deposited on the 4D Nucleome Data Portal (https://data.4dnucleome.org) with the following IDs. iMARGI datasets: H1 control, 4DNESNOJ7HY7; H1 NH4OAc, 4DNESGRI8A8N; H1 FL, 4DNES8B3R3P8; H1 RNase, 4DNESOBRUQ12; HFF, 4DNES9Y1GHK4; K562, 4DNESIKCVASO. Hi-C datasets: H1 control, 4DNESFSCP5L8; H1 NH4OAc, 4DNES2253IBO; H1 FL, 4DNES65I3RQG; H1 RNase, 4DNES4AABNEZ; HFF, 4DNESNMAAN97; K562, 4DNESI7DEJTM. PLAC-seq datasets: H1, 4DNESQMO66LZ; HFF, 4DNESIF5UIQE; K562, 4DNESWX1J3QU.

Field-specific reporting

Please select the one below that is the best fit for your research. If you are not sure, read the appropriate sections before making your selection.

☒ Life sciences ☐ Behavioural & social sciences ☐ Ecological, evolutionary & environmental sciences

For a reference copy of the document with all sections, see nature.com/documents/nr-reporting-summary-flat.pdf

Life sciences study design

All studies must disclose on these points even when the disclosure is negative.

Sample size	No calculation was used to determine sample size. We aimed to generate data from as many replicates as possible.
Data exclusions	No data were excluded.
Replication	For iMARGI and Hi-C biological replicates were included and data were largely consistent.
Randomization	No randomization was used.
Blinding	The authors are not blinded to the data labels.

Reporting for specific materials, systems and methods

We require information from authors about some types of materials, experimental systems and methods used in many studies. Here, indicate whether each material, system or method listed is relevant to your study. If you are not sure if a list item applies to your research, read the appropriate section before selecting a response.

Materials & experimental systems

n/a	Involved in the study	
☐	☒	Antibodies
☐	☒	Eukaryotic cell lines
☒	☐	Palaeontology and archaeology
☒	☐	Animals and other organisms
☒	☐	Human research participants
☒	☐	Clinical data
☒	☐	Dual use research of concern

Methods

n/a	Involved in the study	
☒	☐	ChIP-seq
☒	☐	Flow cytometry
☒	☐	MRI-based neuroimaging

Antibodies

Antibodies used	Mouse monoclonal anti-SC35 primary antibody (Abcam, Cat# ab11826); goat anti-mouse secondary antibody with Alexa Fluor 568 (Invitrogen, Cat# A-11004); rabbit anti-SON primary antibody (Atlas Antibodies, HPA023535); goat anti-rabbit secondary antibody with Alexa Fluor 488 (Invitrogen, Cat# A-11008).
Validation	All the antibodies used in this study have been validated by the vendors as indicated on the websites.

Eukaryotic cell lines

Policy information about cell lines

Cell line source(s)	Human embryonic stem cells (H1), hTert-immortalized human foreskin fibroblasts (HFF), and chronic myelogenous leukemia lymphoblasts (K562) were obtained from the 4D Nucleome (4DN) Cell Repository and cultured following the 4DN Consortium's approved culture protocol for each cell line (https://www.4dnucleome.org/cell-lines.html).<br><br>dCas9-KRAB inducible cells. The doxycycline-inducible dCas9-KRAB H1 ES cell line is generated and karyotyped by the 4D Nucleome Consortium (Danwei Huangfu Laboratory) (https://4dnucleome.org), with TRE-dCas9-KRAB and CAGGS-M2rtTA targeted into the AAVS1 locus.<br><br>HERV-H deletion and insertion cells. The control H9 human ES cells (H9 MLC2v:H2B), HERV-H deletion cell line (H9 MLC2v:H2B HERV2-KO), and HERV-H insertion cell line (H9 MLC2v:H2B HERV2-ins-clone2) were generated by Bing Ren lab and described in reference42.<br><br>RPB1 The auxin-inducible degron 2 cells. The RPB1 auxin-inducible degron 2 cells (HCT116 RPB1-Dox-OsTIR1-mClover-mAID) were generated by Masato Kanemaki lab and described in reference65.
Authentication	The cell lines in the 4DN Cell Repository were established by the 4DN Consortium in collaboration with WiCell and ATCC for providing quality-controlled cells from the identical batch to minimize cell source and culture condition variations. The cell culture protocols were developed by the 4DN Cell Line Working Group and approved by the 4DN Steering Committee.
Mycoplasma contamination	Our lab runs mycoplasma tests on all cells in culture at quarterly basis to ensure no mycoplasma contamination.
Commonly misidentified lines (See ICLAC register)	N/A
