# Supplementary figures and images for: Genome-wide analysis of the interplay between chromatin-associated RNA and 3D genome organization in human cells
Source: Nat Commun. 2023 Oct 16;14:6519. doi: 10.1038/s41467-023-42274-7 (PMC10579264; doi:10.1038/s41467-023-42274-7)

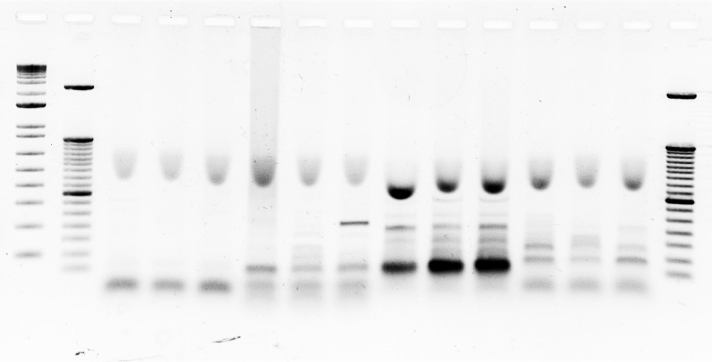

Supplement: Supplementary file 6 — Source Data [file 41467_2023_42274_MOESM6_ESM.zip › SourceData/Figure_4g_raw_uncropped.png]

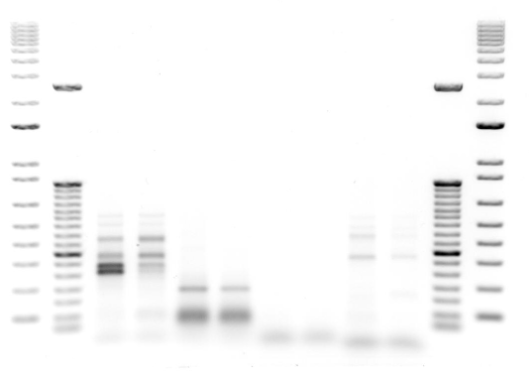

Supplement: Supplementary file 6 — Source Data [file 41467_2023_42274_MOESM6_ESM.zip › SourceData/Figure_3b_raw_uncropped.png]
